# Supplementary material for: Ultrastructural Changes of Blood Cells in Children with Generalized Purulent Peritonitis: A Cross-Sectional and Prospective Study
Source: Children (Basel). 2020 Oct 17;7(10):189. doi: 10.3390/children7100189 (PMC7602975; doi:10.3390/children7100189)
Supplement: Supplementary file 1 [file children-07-00189-s001.pdf]

children

Supplementary Materials

Ultrastructural Changes of Blood Cells in Children With Generalized Purulent Peritonitis: A Cross-Sectional and Prospective Study

Ulyana Halyuk, Olena Lychkovska, Oksana Mota, Vasyl Kovalyshyn, Natalia Kech, Petro Pokotylo, Olena Trutiak, Bożena Zboina, Grzegorz Józef Nowicki and Barbara Ślusarska

Table S1. SIRS assessment results of the children researched.

Patient number	Age [years]	Gender [1-male]	Duration of symptoms [hours]	Temperature [°C]	Heart rate [min]	Respiratory rate [min]
1	8	1	26	38.6	102	27
2	10	0	32	38.1	110	30
3	7	0	42	39	118	29
4	8	1	84	40.3	130	35
5	11	1	48	38.2	117	24
6	11	1	60	38.9	122	33
7	12	0	36	39.5	132	36
8	9	1	36	38.3	118	25
9	9	1	48	38.5	105	27
10	8	0	72	38.2	109	31
11	9	1	60	38.9	128	29
12	10	0	84	39.1	136	27
13	6	0	36	38.5	126	33
14	6	1	36	40.1	144	33
15	12	1	60	39.8	132	31

Table S2. SOFA assessment results of the children researched.

Patient number	Respiration	Coagulation	Liver	Cardiovascular	Catecholamin	Central Nervous System	Renal	SOFA score
	PaO_2/FIO_2 [mmHg]	Platelets [x10³µL]	Bilirubin [mg/dL]	MAP [mmHg]		GCS	Creatinine [mg/dL]	
1	356	140	1.4	68	No	14	1.6	1
2	326	131	1.7	57	No	14	1.4	1
3	322	129	1.8	68	No	13	1.7	1
4	287	98	3.5	82	Yes	12	4.1	2
5	333	139	1.2	55	No	14	1.3	1
6	218	76	4.1	75	Yes	10	3.9	2
7	254	81	3.6	45	Yes	11	4.0	2
8	339	141	1.7	68	No	14	1.5	1
9	396	148	1.3	67	No	14	1.3	1
10	377	140	1.4	66	No	14	1.4	1
11	355	145	1.6	59	No	14	1.3	1
12	339	122	1.4	66	No	14	1.2	1
13	311	118	1.3	63	No	14	1.4	1
14	285	83	4.1	71	Yes	11	2.9	2
15	229	75	5.1	65	Yes	12	3.2	2

Note: PaO_2: partial pressure of oxygen; FIO_2: fraction of inspired oxygen; MAP: mean arterial pressure; GSC: Glasgow Coma Scale.
